# Retrospective Description of Pregnant Women Infected with Severe Acute Respiratory Syndrome Coronavirus 2, France

**DOI:** 10.3201/eid2609.202144

**Published:** 2020-09

**Authors:** Alexandre J. Vivanti, Jérémie Mattern, Christelle Vauloup-Fellous, Jacques Jani, Luc Rigonnot, Larissa El Hachem, Agnès Le Gouez, Céline Desconclois, Imane Ben M’Barek, Jeanne Sibiude, Alexandra Benachi, Olivier Picone, Anne-Gaël Cordier

**Affiliations:** Antoine Béclère Hospital, Paris Saclay University, Clamart, France (A.J. Vivanti, J. Mattern, A. Le Gouez, C. Desconclois, A. Benachi);; Paul Brousse Hospital, Paris Saclay University, Villejuif, France (C. Vauloup-Fellous);; University Hospital Brugmann, Université Libre de Bruxelles, Brussels, Belgium (J. Jani);; Centre Hospitalier Sud-Francilien, Corbeil-Essonnes, France (L. Rigonnot, L. El Hachem);; Bicêtre Hospital, Paris Saclay University, Le Kremlin-Bicêtre, France (I. Ben M’Barek, A.-G. Cordier);; Louis Mourier Hospital, Paris University, Colombes, France (J. Sibiude, O. Picone)

**Keywords:** pregnancy, respiratory infections, severe acute respiratory syndrome coronavirus 2, SARS-CoV-2, SARS, COVID-19, coronavirus disease, zoonoses, viruses, coronavirus, MERS, France

## Abstract

Fix data are available on the management of pregnant women infected with severe acute respiratory syndrome coronavirus 2 (SARS-CoV-2). We conducted a retrospective study of 100 pregnant women with SARS-CoV-2 infection in 4 obstetric units in the Paris metropolitan area of France during March 12–April 13, 2020. Among patients, 52 (52%) were hospitalized, 10 (10%) in intensive care units (ICUs). Women with higher body mass indexes (BMIs; median 30.7 kg/m^2^) were more likely to be hospitalized in ICUs than other women (median BMI 26.2 kg/m^2^). Women hospitalized in ICUs had lower lymphocyte count at diagnosis (median 0.77 × 10^9^ cells/L) than women not hospitalized in ICUs (median lymphocyte count 1.15 × 10^9^ cells/L). All women requiring oxygen >5 L/min were intubated. Clinical and laboratory evaluation of SARS-CoV–2−positive pregnant women at the time of diagnosis can identify patients at risk for ICU hospitalization.

Recent literature from China, Italy, and the United States suggests that pregnant women are not at higher risk for severe forms of coronavirus disease (COVID-19) from infection with severe acute respiratory syndrome (SARS) coronavirus 2 (SARS-CoV-2), contrary to what has been reported with SARS and MERS (*1*–*3*). Nevertheless, 3%–35% of infected pregnant women were hospitalized in intensive care units (ICUs) (*2*,*4*–*7*) and respiratory and hematology anomalies were described, just as in the nonpregnant infected population (*8*). In the third trimester, and especially after 37 weeks’ gestation, the fetal prognosis is driven by maternal clinical tolerance and by whether a cesarean delivery is required. Few cases of vertical transmission have been published (*6*–*9*), and no data are available on the risk factors for such transmission. However, between 24 and 32 weeks’ gestation, the risk for premature birth and the need to reduce its effects on neonatal outcome by giving steroids and magnesium sulfate to the mother complicate decision-making. Little published data are available on the management of SARS-CoV-2−infected pregnant women (*10*). We describe the experience of 4 tertiary referral obstetric units in managing such cases in the Paris metropolitan area of France.

## Materials and Methods

### Study Design and Population

We conducted a retrospective multicenter review of the medical records of all pregnant women with SARS-CoV-2 from March 12–April 13, 2020, in 4 tertiary referral obstetric units in the Paris metropolitan area. Hospitals included in the study were Antoine Béclère, Clamart; Bicêtre Hospital, Le Kremlin Bicêtre; Louis-Mourier, Colombes; and Centre Hospitalier Sud Francilien, Evry. All women in the second and third trimester of pregnancy (>14 weeks’ gestation) had real-time reverse transcription PCR (RT-PCR) testing of respiratory tract samples to detect SARS-CoV-2. Gestational age was calculated according to crown–rump length measurement at the first-trimester scan. Because of the moderate sensitivity of RT-PCR (*11*–*13*), patients with negative results also had computed tomography imaging of the chest, which was considered positive when meeting conventional criteria for SARS-CoV-2 infection (*14*). Patients were considered cured >10 days after the positive diagnosis and without clinical signs for 48 h, or 14 days from the beginning of the disease with only benign signs without hospitalization.

We retrospectively allocated women to subgroups according to location for further care after first assessment. The outpatient follow-up group was defined as infected pregnant women who could return home after assessment in the emergency department. Close outpatient follow-up was undertaken and included a daily call from an obstetrician/gynecologist. In the case of suspected worsening, such as fever, dyspnea, or tachycardia, or obstetric concern, women were asked to return to the hospital for further assessment and hospitalized, if needed. The conventional hospitalization group was defined as women immediately hospitalized after SARS-CoV-2 diagnosis because close medical supervision or noninvasive oxygen therapy with flow rates <3 L/min, was needed. The ICU hospitalization group was defined as women hospitalized in ICUs because of clinical worsening, including respiratory distress or increased oxygen requirement, and the need for continuous medical supervision, noninvasive high-flow oxygen delivery, or invasive mechanical ventilation.

### Sample Collection

Laboratory samples were obtained for RT-PCR and prepared as follows. Nasopharyngeal swabs were obtained following US Centers for Disease Control and Prevention guidelines (*15*). Swabs were placed in Virocult viral transport media (Sigma, https://www.sigmaaldrich.com). All specimens were kept at 4°C and tested within 24 hours.

### RT-PCR

Viral RNA was extracted from 200 µL of clinical samples with the NucliSENS easyMag kit (BioMérieux, https://www.biomerieux.com) and eluted in 100 µL. The RealStar SARS-CoV-2 RT-PCR Kit 1.0 (Altona Diagnostics GmbH, https://www.altona-diagnostics.com) targeting the E gene, specific for lineage B-betacoronavirus, and the S gene, specific for SARS-CoV-2, was used, according to the manufacturer’s recommendations. The assay includes a heterologous amplification system as an internal positive control to identify possible RT-PCR inhibition and to confirm the integrity of the reagents of the kit. Thermal cycling was performed at 55°C for 20 min for reverse transcription, followed by 95°C for 2 min, and then 45 cycles of 95°C for 15 s, 55°C for 45 s, and 72°C for 15 s with an Applied Biosystems ViiA7 instrument (ThermoFisher Scientific, https://www.thermofisher.com). A cycle threshold <40 was considered positive for SARS-CoV-2 RNA.

### Data Collection and Statistical Analysis

We retrospectively collected clinical, laboratory, and imaging data on mothers and newborns from medical records. We performed statistical analyses by using GraphPad Prism version 8.0.0 (GraphPad Software, https://www.graphpad.com). We used a 2-tailed Mann-Whitney U test for statistical analysis of continuous variables and Fisher exact test for statistical analysis of categorical variables. We expressed continuous variables as median with interquartile range (IQR) and categorical variables as number (percentage). We considered p<0.05 statistically significant.

This study was approved by the institutional review board of the French College of Obstetricians and Gynecologists (approval no. CEROG OBS-2020–0402). All data were de-identified to ensure patient privacy and confidentiality.

## Results

### Maternal Characteristics and Signs and Symptoms at Diagnosis

During March 12–April 13, 2020, a total of 240 women were tested for SARS-CoV-2 infection during pregnancy or the early postpartum period because of relevant symptoms (Figure). Among them, 100 (42%) were considered infected; 99 (41%) had a positive RT-PCR, and 1 woman with a negative RT-PCR test was considered positive because computed tomography imaging of the chest was compatible with SARS-CoV-2 infection. The median age of the infected women was 33.7 years (range 29–36.7 years); 81 (81%) were tested after 24 weeks’ gestation, and 18 (18%) were tested between 14 and 24 weeks’ gestation. One asymptomatic patient was tested 2 days postpartum because of an isolated increase in activated partial thromboplastin time (aPPT; ratio 1.40), but signs of primary coagulopathy or consumption were not noted, and the patient’s platelet count was 154 × 10^9^/L, prothrombin time ratio was 100%, and fibrinogen activity was 5.5 g/L. 

**Figure:**
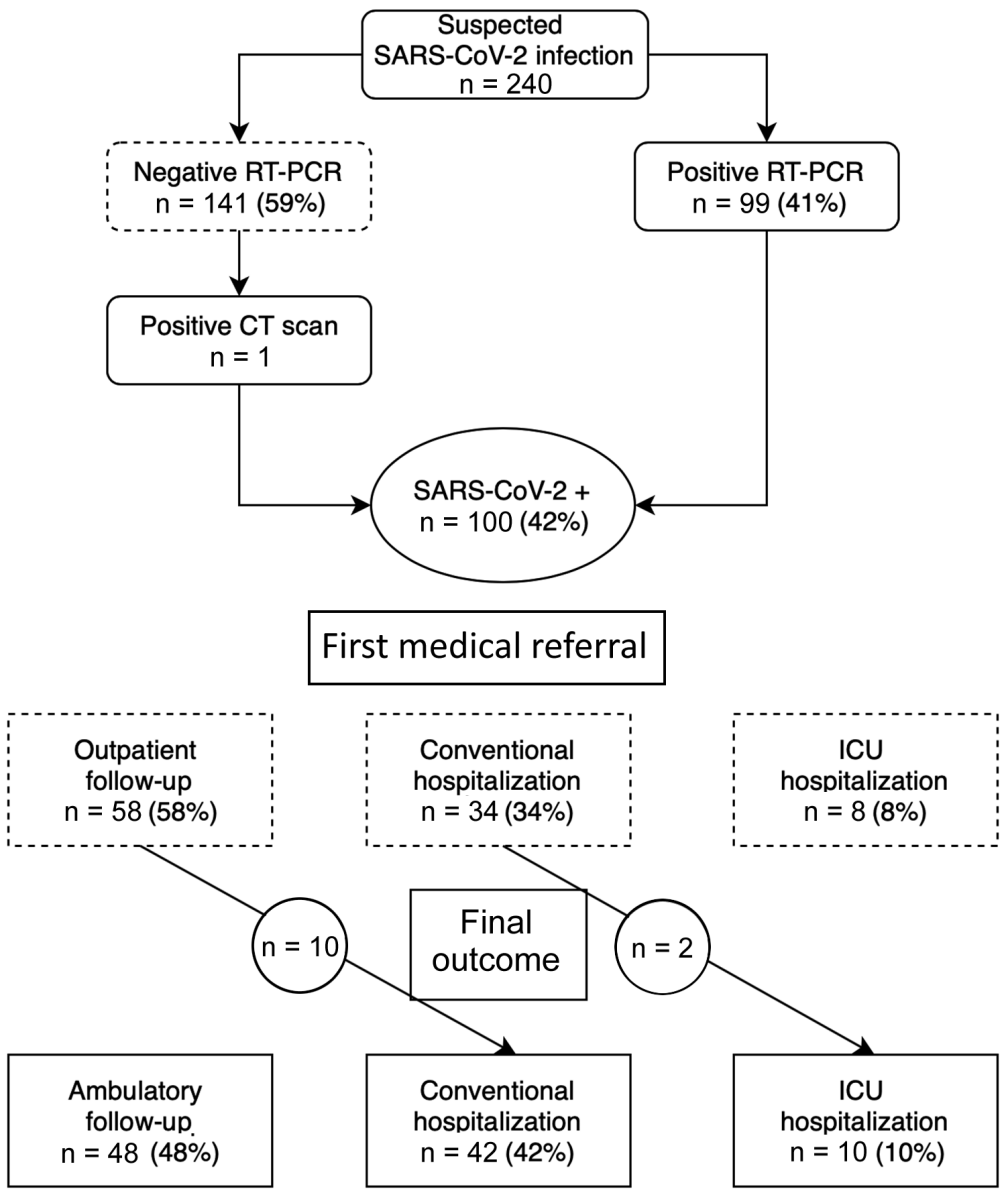
Flowchart of study data showing status and medical referral in a cohort of pregnant women with SARS-CoV-2 infection, France. CT, computed tomography; RT-PCR, reverse transcription PCR; SARS-CoV-2, severe acute respiratory syndrome coronavirus 2.

A few patients had underlying conditions, including 9 (9%) with asthma, 7 (7%) with diabetes mellitus, and 6 (6%) with chronic high blood pressure. Median body mass index (BMI) was 27.0 kg/m^2^ (IQR 23.5–30.6 kg/m^2^). At diagnosis, all but 1 woman experienced symptoms compatible with SARS-CoV-2 infection; 80 (80%) had cough, 62 (62%) had fever, 30 (30%) had dyspnea, 26 (26%) had myalgia, 16 (16%) had anosmia, and 10 (10%) had gastrointestinal symptoms. None reported rash (Table 1).

**Table 1:** Baseline characteristics at diagnosis for pregnant women infected with sever acute respiratory syndrome 2, France*

Maternal and obstetric characteristics	Value
Total patients	100
Median age, y (IQR)	33.7 (29–36.7)
Median gravidity (IQR)	3 (1.8–4)
Median parity (IQR)	1 (0–3)
Median BMI, kg/m^2^ (IQR)	27.0 (23.5–30.6)
Preexisting conditions	
Diabetes mellitus	7 (7)
Chronic high blood pressure	6 (6)
Tobacco use	2 (2)
Asthma	9 (9)
Median gestational age at diagnosis, wk (IQR)	31.3 (25.6–35.6)
14–24	18 (18)
25–32	41 (41)
33–37	20 (20)
>37	20 (20)
Early postpartum	1 (1)
Signs and symptoms	
Fever	62 (62)
Cough	80 (80)
Dyspnea	30 (30)
Myalgia	26 (26)
Anosmia	16 (16)
Sore throat	9 (9)
Diarrhea or vomiting	10 (10)
Rash	0
Other signs	13 (13)

*Values are no. (%) except as indicated. BMI, body mass index; IQR, interquartile range.

After a first evaluation, 58 (58%) of the infected women were eligible for close outpatient follow-up; 10 (17%) were hospitalized during later follow-up. Forty-two (42%) women were immediately hospitalized, 8 (19%) in ICUs and 34 (80.9%) in conventional wards. Later, 2 women were switched from conventional hospitalization to the ICU because of increased respiratory symptoms. Among all patients, 32 (32%) required oxygen therapy. At the end of the study period, 48 (48%) patients received outpatient follow-up only, and 52 (52%) were hospitalized, 42 in conventional wards and 10 in ICUs. 

We found that women with a high BMI (30.7 kg/m^2^ [IQR 29.8–33.1 kg/m^2^]) were more likely to be hospitalized in ICUs than women with lower BMIs (26.2 kg/m^2^ [IQR 23–29.7 kg/m^2^]; p = 0.003). We noted no statistically significant difference in maternal age, gravidity, parity, gestational age at diagnosis, or preexisting medical conditions among patients admitted to ICUs (Table 2). We performed a similar comparison between women with outpatient follow-up (BMI 26.2 kg/m^2^ [IQR 22.9–29.9 kg/m^2^]) and women who required hospitalization (BMI 30.7 kg/m^2^ [IQR 24.5–30.9 kg/m^2^]). We noted BMI also was the only maternal baseline characteristic with a statistically significant difference (p = 0.003) between the 2 groups (Appendix). No maternal thromboembolic event was noted during the study period.

**Table 2:** Maternal and obstetric characteristics according to medical referral for pregnant women with severe acute respiratory syndrome 2 infection, France*

Characteristics	Non-ICU hospitalization, n = 90	ICU hospitalization, n = 10	p value
Median age, y (IQR)	33.2 (29.1–36.7)	33.6 (28.3–34.3)	0.89
Median BMI, kg/m^2^ (IQR)	26.2 (23–29.7)	30.7 (29.8–33.1)	0.003
Underlying conditions, no. (%)			
Diabetes mellitus	7 (8)	0	1
Chronic high blood pressure	5 (6)	1 (10)	0.48
Tobacco use	2 (2)	0	1
Asthma	7 (8)	2 (20)	0.22
Median gestational age at diagnosis, wks (IQR)	31.3 (25–35.6)	28.5 (26.9–34.2)	0.78

*ICU, intensive care unit; IQR, interquartile range

### Laboratory Parameters at SARS-CoV-2 Diagnosis

We analyzed laboratory parameters at diagnosis for maternal medical care (Table 3). Lymphocyte count at diagnosis was lower in women hospitalized in ICUs (0.77 × 10^9^ cells/L [IQR 0.7–1× 10^9^ cells/L]) than in women in the conventional hospitalization or outpatient follow-up groups (1.15 × 10^9^ cells/L [IQR 0.9–1.6 × 10^9^ cells/L]; p = 0.01). Moreover, the proportion of women with lymphocytopenia at diagnosis also was much higher in the ICU group (89%) than in the rest of the cohort (36%; p = 0.008). Hemoglobin count at diagnosis was lower in women who needed hospitalization in ICUs (9.8 g/dL [IQR 9.3–11.3 g/dL]) than in the rest of the cohort (11.4 g/dL [IQR 10.5–12.2 g/dL]; p = 0.02). We did not detect any statistically significant differences between the 3 groups for white cell count, prothrombin time, aPPT, fibrinogen activity, alanine aminotransferase, aspartate aminotransferase, C-reactive protein, or creatinine. We performed a similar comparison between women with outpatient follow-up and women who required hospitalization and found no statistically significant between-group differences in any laboratory parameter (Appendix Table 2). We assessed laboratory parameters at diagnosis and oxygen therapy requirements and noted that women with lymphocytopenia and prolonged aPPT at diagnosis were more likely to need an oxygen therapy (Appendix Table 3). Similarly, we noted lower lymphocyte counts, increased aPPT ratios, and increased C-reactive protein levels for women who required oxygen therapy than for the others.

**Table 3:** Laboratory parameters at diagnosis according to medical referral for pregnant women with severe acute respiratory syndrome 2 infection, France*

Laboratory findings	Non-ICU hospitalization, n = 90			ICU hospitalization, n = 10		p value
	Median (IQR)	No. (%)		Median (IQR)	No. (%)	
Hemoglobin, g/dL	11.4 (10.5–12.2)	64 (66.7)		9.8 (9.3–11.3)	9 (90)	0.02
Platelet count, × 10^9^/L	230 (162–273)	63 (70.0)		205 (164–271)	9 (90)	0.98
Leukocyte count, × 10^9^ cells/L	7.2 (5.4–8.9)	63 (70.0)		6.6 (6.1–7.2)	9 (90)	0.68
Lymphocyte count, × 10^9^ cells/L	1.15 (0.9–1.6)	58 (64.4)		0.77 (0.7–1)	9 (90)	0.01
Lymphocytopenia, <1.00 × 10^9^ cells/L	NA	21/58 (36.2)†		NA	8/9 (88.9)†	0.008
Prothrombin time, %	100 (99–100)	53 (58.9)		100 (100–100)	7 (70)	0.61
aPPT, ratio	1.06 (1–1.2)	52 (57.8)		1.12 (1–1.4)	7 (70)	0.16
Prolonged aPPT ratio (>1.20)	NA	13/53 (24.5)†		NA	3/7 (43)†	0.38
Fibrinogen activity, g/L	4.8 (4–5.8)	45 (50.0)		5.1 (4.5–5.5)	6 (60)	0.73
AST, U/L	25 (20–35)	48 (53.3)		30 (22–59)	8 (80)	0.38
ALT, U/L	17 (11–32)	49 (54.4)		19 (12–48)	8 (80)	0.46
C-reactive protein, mg/L	23 (9–42)	53 (58.9)		27 (22–108)	8 (80)	0.15
Creatinine, μmol/L	47 (41–57)	45 (50.0)		50 (38–55)	7 (70)	0.94

*ALT, alanine aminotransferase; aPPT, activated partial thromboplastin time; AST, aspartate aminotransferase; ICU, intensive care unit; IQR, interquartile range; NA, not applicable. †Per available results.

### Obstetric and Neonatal Outcomes

At the end of the study period, 33 women (33%) had delivered 36 neonates, including 3 twin deliveries. Median gestational age of neonates was 37.9 weeks (IQR 35–40.1 weeks) (Table 4). Deliveries in SARS-CoV-2−infected women represented 2.4% of the 1,362 deliveries in the 4 hospitals. Preterm births, those at <37 weeks’ gestation, represented 39% of the whole cohort; the median interval between SARS-CoV-2 diagnosis and delivery was 3 days (IQR 1–9 days). No stillbirths or miscarriages occurred among the study population. Among deliveries, 16 (48%) were cesarean deliveries, 13 (36%) of which were because of SARS-CoV-2 infection, 12 because of maternal respiratory distress, and 1 because of major coagulopathy. All women who delivered before 32 weeks’ gestation were given antenatal magnesium sulfate therapy. All but 1 of the women who delivered before 34 weeks’ gestation were given antenatal corticosteroid therapy (2 doses of betamethasone 12 mg given intramuscularly 24 hours apart).

**Table 4:** Obstetric and neonatal outcomes for 100 pregnant women with severe acute respiratory syndrome 2 infection, France*

Obstetric outcomes	Value
Ongoing pregnancies	67 (67.0)
Stillbirths or miscarriages	0
Deliveries	33 (33.0)
Median days between SARS-CoV-2 diagnosis and delivery (IQR)	3 (1–9)
Delivery mode	
Vaginal	17 (51.5)
Spontaneous labor	9 (52.9)
Induced labor, reason	8 (47.1)
Respiratory degradation	4 (50.0)
Preeclampsia	1 (12.5)
Intrahepatic cholestasis	1 (12.5)
Reduced fetal movements	1 (12.5)
Premature rupture of membranes	1 (12.5)
Caesarean delivery, reason	16 (48.5)
Respiratory distress	12 (36.4)
Major coagulopathy	1 (6.3)
Severe preeclampsia	1 (6.3)
Non-reassuring fetal heart rate	1 (6.3)
Definitive cervicoisthmic cerclage	1 (6.3)
Gestational age at birth, wk, median (IQR)	37.9 (35–40.1)
>37	20 (60.6)
32–36	13 (39.4)
24–31	7 (21.2)
Twin pregnancies	3 (3.0)
Neonates†	36
Birthweight z-score, g, median (IQR)	0.15 (–0.75 to 0.64)
<10th percentile	1 (3)
Apgar score <7	
1 min	8 (22)
5 min	4 (11)
10 min	1 (3)
Umbilical arterial pH, median (IQR)	7.26 (7.24–7.29)
Neonatal intubation	6 (17)
NICU hospitalization	10 (28)
Neonatal death	0
Neonate SARS-CoV-2–positive	1 (3)

*Values are no. (%) except where indicated. IQR, interquartile range; SARS-CoV-2, severe acute respiratory syndrome coronavirus 2. †33 women gave birth, including 3 sets of twins.

Only 1 neonate had a birthweight below the 10th percentile; 10 were hospitalized in the neonatal ICU (NICU) because of prematurity. No neonatal acidosis was noted; median umbilical arterial pH was 7.26 (IQR 7.24–7.29), even for most severe maternal cases. 

All the neonates were tested for SARS-CoV-2 infection. Only 1 neonate tested positive for SARS-CoV-2; his mother had no severe clinical symptoms but experienced cough and fever at 35 weeks’ gestation. At admission, her laboratory workup showed mild thrombocytopenia and prolonged aPPT. Her symptoms improved rapidly during early postpartum. The infant did not require oxygen, but results of RT-PCR testing on nasopharyngeal secretions were positive. He did not show signs of respiratory illness.

Among 3 twin pregnancies, the mother’s SARS-CoV-2 diagnosis was made between 31.4 and 35.4 weeks’ gestation; 1 of the mothers was severely obese (BMI 49 kg/m^2^). At the time of diagnosis, 1 patient had severe lymphocytopenia (0.35 × 10^9^ cells/L) and another had prolonged aPTT (ratio 1.22). Two women were hospitalized because of dyspnea; both had cesarean deliveries, at 31.7 and 35.4 weeks’ gestation, due to increased respiratory complications, but neither was admitted to the ICU. The third had a vaginal delivery at 37 weeks’ gestation. One pair of twins was hospitalized in the NICU because of prematurity. Neonatal acidosis was not observed in any cases. 

### Pregnant Women in ICUs

At the end of the study period, 10 (10%) patients had been admitted to ICUs: 7 during prenatal period (median gestational age at admission 27.9 weeks [IQR 27.2–28.8 weeks]) and 3 during early postpartum, <3 days postpartum (Table 5). Among patients admitted to ICUs, 9 (90%) required intubation after oxygen requirements reached >5 L/min; the mean interval between increased oxygen need and intubation was 28.7 hours (SD + 49.9 h). Six patients had acute respiratory distress syndrome (ARDS); 5 were treated with drug regimen, 3 with lopinavir, and 2 with hydroxychloroquine; 1 was placed in the prone position for ARDS. The average length of stay in the ICU was 9.1 + 5.7 days.

**Table 5:** Maternal outcomes of 10 pregnant women admitted to the intensive unit with severe acute respiratory syndrome coronavirus 2, France*

ID	Age, y	BMI, kg/m^2^	Underlying conditions				Time, h		Intubation, d	ICU stay, d	Drug regimens	Complications
				GA, wk, d			From O_2_ >5 L/min to intubation	From intubation to delivery				
				At diagnosis	At intubation							
1	30.9†	35.8	NA	28, 5	29, 0		15.5	0	10	12	Lopinavir	NA
2	26.5†	29.9	NA	38, 1	POD 2		7	NA	15	16	Hydroxy	Surgical site infection
3	24.9†	25.7	Asthma	28, 5	30, 1		10	0	11	13	Lopinavir	Iatrogenic pancreatitis
4	32.6†	41.8	Hyper-tension	26, 0	26, 1		10.5	7	36	38	Lopinavir	Refractory hypoxemia
5	33.6	30.8	NA	26, 6	27, 6		5.5	0	2	3	NA	NA
6	39.4	31.3	Hashimoto thyroiditis	40, 5	POD 8		25	NA	4	5	NA	NA
7	33.1†	30.5	NA	23, 5	23, 5		1	Ongoing pregnancy	13	14	Hydroxy	Iatrogenic transient hepatitis
8	33.4	29.7	NA	28, 2	NA		NA	NA	NA	3	NA	NA
9	26.1	33.7	Asthma	36, 0	POD 1		24	NA	1	2	NA	NA
10	42.1†	29.3	NA	26, 6	27, 2		160	0	13	14	NA	NA

*BMI, body mass index; GA, gestational age; Hydroxy, hydroxychloroquine; ID, patient identification; NA, not applicable; POD, postoperative day.  †Patients who experienced acute respiratory distress syndrome before intubation.

Among women hospitalized in ICUs, 8 had cesarean deliveries because of rapid respiratory worsening: 5 before 32 weeks’ gestation, 1 between 32 and 37 weeks’ gestation, and 2 after 37 weeks’ gestation (Table 6). During their ICU stays, 3 women had complications: 1 had surgical site infection after cesarean delivery, 1 had iatrogenic pancreatitis attributed to lopinavir with Balthazar grade C, and 1 had iatrogenic and transient hepatitis attributed to hydroxychloroquine. No maternal deaths were noted. Among neonates delivered in this group, no acidosis or birthweight below the 10th percentile were noted. Five neonates were hospitalized in NICUs because of prematurity; 1 died at 7 days of age because of prematurity and bacterial sepsis (Tables 5, 6). Two women left the ICU with ongoing pregnancies.

**Table 6:** Obstetric and neonatal outcomes for pregnant women admitted to an intensive care unit with severe acute respiratory syndrome coronavirus 2, France*

ID	Prenatal corticosteroid, GA, wk, d†	Prenatal magnesium sulfate, GA, wk, d†	Time from diagnosis to delivery, d	Mode of delivery	GA at birth, wk, d	Birthweight, g (z-score)	5-min Apgar score	Umbilical arterial pH	Neonatal intubation	NICU
1	NA	29	2	Cesarean	29	1,400 (1.10)	7	7.26	Y	Y
2	NA	NA	8	Cesarean	39, 1	3,290 (0.06)	10	7.21	N	N
3	30, 1	30+1	11	Cesarean	30, 2	1,500 (0.75)	8	7.25	Y	Y
4	26, 4	26+4	7	Cesarean	27, 1	1,010 (0.82)	1	7.28	Y	Y
5	26, 1	26, 4	7	Cesarean	27, 6	890 (−0.83)	3	7.24	Y	Y
6	NA	NA	1	Cesarean	40, 6	3,570 (0.18)	3	ND	N	N
7	NA	23, 5	Ongoing pregnancy	NA	NA	NA	NA	NA	NA	NA
8	NA	29, 4	Ongoing pregnancy	NA	NA	NA	NA	NA	NA	NA
9	NA	NA	1	Cesarean	36, 1	2,940 (0.49)	10	ND	N	N
10	26, 6	27, 1	3	Cesarean	27, 2	1,065 (1.12)	10	7.27	Y	Y

*All neonates tested negative for SARS-CoV-2. GA, gestational age; NA, not applicable; ND, not done; SARS-CoV-2, severe acute respiratory syndrome coronavirus 2. †At time of drug administration.

## Discussion

We report detailed experience managing 100 patients infected with SARS-CoV-2 in tertiary referral obstetric units during the COVID-19 pandemic in France. Nearly half (48/100) received close outpatient follow-up without any clinically significant events. The other 52 were hospitalized for monitoring or oxygen therapy, including 10 (10%) who had critical infections and required hospitalization in ICUs. Lymphocytopenia, anemia, and need for oxygen flow >5 L/min at the time of diagnosis seem to be associated with a critical infection.

The management of pregnant women with SARS-CoV-2 is a particularly critical issue in tertiary referral obstetric units. In our cohort, 52% of patients were hospitalized and 9% required invasive ventilation. The rate of severe and critical forms of COVID-19 reported among this group is higher than previously reported (*5*,*6*,*17*–*21*), which can be explained by the general admissions to tertiary referral obstetric units. Tertiary obstetric units accept women with high-risk pregnancies and referrals from other maternity hospitals that lack technical platforms needed to support them. The 4 centers in our study offer adult and neonatal resuscitation, enabling optimal maternal management. Clinicians must weigh the continuation of the pregnancy against all the risks associated with premature birth that can lead to neonatal death. Having an adult ICU in the same facility as the maternity ward makes it possible to continue the pregnancy under conditions that seem acceptable.

Maternal and fetal clinical assessment at the time of diagnosis is essential for appropriate medical referral. Systematic laboratory tests, including hemoglobin level, blood count, hemostasis, and inflammatory evaluation, at the time of SARS-CoV-2 diagnosis in pregnant women could help to determine the level of risk for progression to an unfavorable form. Lymphocytopenia, increased C-reactive protein, and increased aPPT have an unfavorable prognostic value that could lead to an increased risk for severe COVID-19 forms in nonpregnant adults (*22*–*25*). Our study underlines the need to consider the lymphocyte count in the choice of medical approach; when counts are acceptable, outpatient management can be safely considered. However, close telemonitoring is required. In our cohort, 17% of patients followed up on an outpatient basis subsequently required hospitalization.

During early pregnancy (<32 weeks’ gestation), clinicians tried to continue the pregnancy because of the neonatal risks associated with premature birth. ICU hospitalization alone was not a criterion for delivery. However, analysis of our cohort data shows that in all pregnant women, a need for increasing oxygen flow rate to >5 L/min was a signal for invasive ventilation. All but 1 patient on invasive ventilation required cesarean delivery due to ventilatory instability. Antenatal corticosteroid therapy before 34 weeks’ gestation and a neuroprotective course of magnesium sulfate before 32 weeks’ gestation appears to be safe and appropriate when oxygen requirements increase. An average interval of >24 hours between the increase in oxygen flow rate to >5 L/min and invasive ventilation enables the administration of >1 of 2 recommended doses of corticosteroids and a complete course of magnesium sulfate.

Our study reports clinical and laboratory data at the time of diagnosis used to identify prognostic factors associated with an adverse outcome in pregnant women infected with SARS-CoV-2. Our findings can help clinicians around the world combat the pandemic. 

Our study has several limitations. First, although we wanted to identify prognostic factors associated with adverse outcomes, our sample size of patients admitted to the ICU was too small to perform a robust multivariate analysis; our results are purely descriptive and not predictive. Second, our study used clinical and laboratory data only at the time of diagnosis, and we did not evaluate the effect of subsequent laboratory and clinical features. Finally, our study was retrospective and had missing data values.

Among our cohort, preterm births (<37 weeks’ gestation) accounted for 39% of all deliveries and the cesarean delivery rate was 48%. However, the preterm birth and cesarean delivery rates we report could be lower once all the women with SARS-CoV-2 infections have given birth and full information becomes available. 

Specific information on pregnant women receiving care for COVID-19 is still lacking, and literature from China reports low infection rates in this population. Tertiary referral obstetric units with a maternal ICU play a major role in the management of symptomatic pregnant women. In addition to maternal respiratory symptoms, neonatal conditions related to spontaneous or induced prematurity in relation to SARS-CoV-2 infection must be considered. In our study, careful clinical and laboratory evaluation at the time of diagnosis enabled safe outpatient monitoring for almost half of the pregnant women. Further investigations are required to assess the true risks associated with SARS-CoV-2 infection during pregnancy.

Appendix Additional information on maternal and obstetrics characteristics and laboratory findings for 100 pregnant women infected with severe acute respiratory syndrome coronavirus 2, France.
