# Supplementary material for: Retrospective Description of Pregnant Women Infected with Severe Acute Respiratory Syndrome Coronavirus 2, France
Source: Emerg Infect Dis. 2020 Sep;26(9):2069–76. doi: 10.3201/eid2609.202144 (PMC7454086; doi:10.3201/eid2609.202144)
Supplement: Appendix — Additional information on maternal and obstetrics characteristics and laboratory findings for 100 pregnant women infected with severe acute respiratory syndrome coronavirus 2, France. [file 20-2144-Techapp-s1.pdf]

Article DOI: https://doi.org/10.3201/eid2609.202144

Retrospective Description of Pregnant Women Infected with Severe Acute Respiratory Syndrome Coronavirus 2, France

Appendix

Appendix Table 1. Maternal and obstetric characteristics according to medical referral for pregnant women with severe acute respiratory syndrome coronavirus 2 infection

Characteristics	Outpatient follow-up, n = 48	Hospitalization, n = 52	p value
Age, y; median (IQR)	33.7 (29–37.6)	33.0 (29–35.5)	0.89
BMI, kg/m^2; median (IQR)	26.2 (22.9–29.9)	30.7 (24.5–30.9)	0.003
History of preexisting condition, no. (%)			
Diabetes mellitus	2 (4)	5 (10)	0.44
Chronic high blood pressure	2 (4)	4 (8)	0.68
Tobacco use	0	2 (4)	0.50
Asthma	4 (8)	5 (10)	0.86
Gestational age at diagnosis, wks; median (IQR)	31.6 (24.4–36.6)	30.6 (26.8–35)	0.89

*BMI, body mass index; IQR, interquartile range.

Appendix Table 2. Laboratory parameters at severe acute respiratory syndrome coronavirus 2 diagnosis for 100 pregnant women, France*

Laboratory parameters†	Outpatient follow-up, n = 48	Hospitalization, n = 52	p value
Hemoglobin, g/dL	11.0 (10.5–11.7); 27/48	11.5 (10.2–12.3); 46/52	0.37
Platelet count, ×10^9/L	239 (174–298); 27/48	210 (161–269); 45/52	0.19
White cell count, ×10^9/L	7.2 (5.7–8.8); 27/48	6.7 (5.4–8.9); 45/52	0.63
Lymphocyte count, ×10^9/L	1.22 (0.9–1.8); 24/48	1.04 (0.8–1.4); 43/52	0.23
Lymphocytopenia (<1.00 ×10^9/L), no./number of tests (%)	8/24 (33)	21/43 (49)	0.30
Prothrombin time, %	100 (95–100); 20/48	100 (100–100); 40/42	0.20
aPPT, ratio	0.99 (0.9–1.2); 20/48	1.12 (1–1.3); 39/52	0.09
Prolonged aPPT ratio (≥1.20), no./number of tests (%)	3/20 (15)	13/39 (33)	0.22
Fibrinogen activity, g/L	4.7 (3.8–5.8); 17/48	5.0 (4.2–5.7); 34/52	0.46
AST, U/L	26 (20–34); 15/48	25 (20–36); 41/52	0.81
ALT, U/L	13 (11–23); 16/48	17 (11–34); 41/52	0.68
C-reactive protein, mg/L	11 (5–35); 18/48	25 (17–45); 43/52	0.09
Creatinine, µmol/L	47 (40–60); 15/48	47 (42–56); 37/52	0.69

*ALT, alanine aminotransferase; aPPT, activated partial thromboplastin time; AST, aspartate aminotransferase; IQR, interquartile range.
†All results reported as median (IQR), except where noted, with the ratio of available data/total number of patients for each group.

Appendix Table 3. Laboratory parameters at severe acute respiratory syndrome coronavirus 2 diagnosis for 100 pregnant women, according to oxygen therapy requirement, France*

Laboratory parameters†	No oxygen therapy, n = 68	Oxygen therapy, n = 32	p value
Hemoglobin, g/dL	11.5 (10.4–12.1); 46/68	11.0 (9.8–11.8); 27/32	0.15
Platelet count, ×10^9/L	238 (168–278); 46/68	203 (161–272); 26/32	0.44
White cell count, ×10^9/L	7.1 (5.6–8.9); 46/68	6.6 (5–9.1); 26/32	0.78
Lymphocyte count, ×10^9/L	1.30 (1.0–1.6); 42/68	0.92 (0.7–1.1); 25/32	0.008
Lymphocytopenia (<1.00 ×10^9/L), no./number of tests (%)	12/42 (29)	17/25 (68)	0.002
Prothrombin time, %	100 (96–100); 38/68	100 (100–100); 22/32	0.09
aPPT, ratio	1.05 (0.9–1.2); 37/68	1.17 (1.0–1.3); 22/32	0.02
Prolonged aPPT ratio (≥1.20), no./number of tests (%)	5/37 (13)	11/22 (50)	0.005
Fibrinogen activity, g/L	5.2 (4.1–5.9); 31/68	4.8 (4.3–5.4); 20/32	0.83
AST, U/L	26 (20–35); 33/68	24 (19–41); 23/32	0.98
ALT, U/L	18 (11–32); 34/68	15 (10–34); 23/32	0.49
C-reactive protein, mg/L	21 (4–5.8); 36/68	27 (17–82); 25/32	0.03
Creatinine, µmol/L	47 (40–57); 31/68	46 (42–57); 21/32	0.6

Appendix Table 3. Laboratory parameters at severe acute respiratory syndrome coronavirus 2 diagnosis for 100 pregnant women, according to oxygen therapy requirement, France*

*ALT, alanine aminotransferase; aPPT, activated partial thromboplastin time; AST, aspartate aminotransferase; IQR, interquartile range.
†All results reported as median (IQR), except where noted, with the ratio of available data/total number of patients for each group.
